# Antioxidant, Antinociceptive, and Anti-Inflammatory Activities from *Actinidia callosa* var. *callosa In Vitro* and *In Vivo*

**DOI:** 10.1155/2012/129152

**Published:** 2012-11-18

**Authors:** Jung-Chun Liao, Jeng-Shyan Deng, Ying-Chih Lin, Chao-Ying Lee, Min-Min Lee, Wen-Chi Hou, Shyh-Shyun Huang, Guan-Jhong Huang

**Affiliations:** ^1^School of Pharmacy, College of Pharmacy, China Medical University, Taichung 404, Taiwan; ^2^Department of Health and Nutrition Biotechnology, Asia University, Taichung 413, Taiwan; ^3^Department of Optometry, Jen-Teh Junior College of Medicine, Nursing and Management, Miaoli 356, Taiwan; ^4^Graduate Institute of Pharmacognosy, Taipei Medical University, Taipei 250, Taiwan; ^5^Department of Chinese Pharmaceutical Sciences and Chinese Medicine Resources, College of Pharmacy, China Medical University, Taichung 404, Taiwan

## Abstract

*Actinidia callosa* var. *callosa* has been widely used to treat antipyretic, analgesic, anti-inflammation, abdominal pain, and fever in Taiwan. The aim of this study was to evaluate the antioxidant, antinociceptive, and anti-inflammatory lipopolysaccharide-(LPS-)induced nitric oxide (NO) production in RAW264.7 macrophages and pawedema induced by **λ**-carrageenan activities of the methanol extract from *A. callosa*. In HPLC analysis, the fingerprint chromatogram of ethyl-acetate fraction of *A. callosa* (EAAC) was established. EAAC showed the highest TEAC and DPPH radical scavenging activities, respectively. We evaluated that EAAC and the reference compound of catechin and caffeic acid decreased the LPS-induced NO production in RAW264.7 cells. Treatment of male ICR mice with EAAC significantly inhibited the numbers of acetic acid-induced writhing response and the formalin-induced pain in the late phase. Administration of EAAC showed a concentration-dependent inhibition on paw edema development after Carr treatment in mice. Anti-inflammatory mechanisms of EAAC might be correlated to the expression of inducible nitric oxide synthase (iNOS), cyclooxygenase-2 (COX-2), and heme oxygenase-1 (HO-1) *in vitro* and *in vivo*. Overall, the results showed that EAAC demonstrated antioxidant, antinociceptive, and anti-inflammatory activity, which supports previous claims of the traditional use for inflammation and pain.

## 1. Introduction

Excess reactive oxygen species (ROS) tend to attack susceptible bimolecular causing oxidative imbalance of the antioxidant system. The resulting oxidative stress may lead to aging, inflammation, and other chronic diseases. It has also been reported that a wide array of diseases, ranging from coronary heart disease to cancer, are caused by inflammation [1]. The medicinal plants are relevant in developing of the world as sources of drugs or herbal extracts for various chemotherapeutic purposes. Several anti-inflammatory, neuroprotective, and hepatoprotective drugs have recently been shown to have an antioxidant and/or antiradical scavenging mechanism as part of their activity [2]. 

Inflammation has been shown to be associated with a number of diseases, including rheumatoid arthritis, chronic asthma, multiples sclerosis, inflammatory bowel disease, and psoriasis which are common worldwide [3]. During inflammation, high levels of ROS were also produced to exert a defense against pathogens. NO production is mainly catalyzed by nitric oxide synthase (NOS) which exists in three isoforms, neuronal NOS (nNOS), endothelial NOS (eNOS), and inducible NOS (iNOS). eNOS and nNOS are constitutively expressed and play an important role in normal physiological activities [4]. The iNOS-mediated NO production can promote pathological inflammation. Therefore, selective inhibition on iNOS activity has been established as a therapeutic approach for treating inflammation [5]. In addition, heme oxygenase-1 (HO-1) is believed to play a cytoprotective role in a variety of pathological models such as inflammation [6]. The anti-inflammatory properties of HO-1 are related to the inhibition of adhesion molecule expression and reduction of oxidative stress, while exogenous carbon monoxide decreases the production of inflammatory mediators such as NO and tumor necrosis factor (TNF-*α*) [7].

*Actinidia callosa *var. *callosa *(Actinidiaceae; AC) is a deciduous tuberous plant, which is distributed in oriental countries and the stem of plant has been traditionally used for the treatment of antipyretic, anti-inflammatory, and analgesic effects [8]. *Actinidia *species had showed some pharmacological effects such as anti-inflammatory activity from the fruit of *Actinidia polygama* [9], antitumor, and immunomodulatory activity from the roots of *Actinidia eriantha* [10]. In this study, we have showed some physiological effects, but there are no studies focusing on its inhibitory effects on the antioxidant, analgesic activities, and the mechanism of anti-inflammatory activities of the methanolic extracts of *Actinidia callosa *var. *callosa* (MAC) and fractions in cell and animal models. Consequently, the objective of the present study is to determine the therapeutical effects of AC against antioxidant, analgesic, and anti-inflammatory activities *in vitro* and *in vivo*. 

## 2. Materials and Methods

### 2.1. Materials

Lipopolysaccharide (LPS, *Escherichia coli *O127 : B8), 1,1-Diphenyl-2-picrylhydrazyl (DPPH), *N*-(1-naphthyl) ethylenediamine dihydrochloride, sulfanilamide, 2,2′-azino-bis (3-ethylbenzothiazoline-6-sulphonic acid) (ABTS), thiobarbituric acid (TBA), 3-[4,5-dimethyl-thiazol-2-yl]-2,5-diphenyl tetrazolium bromide (MTT), *λ*-Carrageenan (Carr), indomethacin (Indo), and other chemical reagents were purchased from Sigma-Aldrich (St. Louis, MO, USA). Plant materials were collected from Taichung country in Taiwan. They were identified and authenticated by Dr. Yuan-Shiun Chang, Professor, Department of Chinese Pharmaceutical Sciences and Chinese Medicine Resources, College of Pharmacy, China Medical University. 

### 2.2. Extraction and Fractionation

The coarse powder of *A. callosa *var. *callosa* (AC) (2 kg) was extracted with methanol three times. The extract was evaporated under reduced pressure using a Rotavapor, and then stored under light protection. A yield equivalent to 6.51% of the original weight was obtained. Next, MAC (130.2 g) was dissolved and suspended in 100 mL of water in a separatory funnel prior to being partitioned in sequence with *n-*hexane, ethyl acetate, and *n-*butanol (800 mL each for three times). Under reduced pressure, fractions were yielded and collected: *n-*hexane fraction (17.79 g, 13.66%), ethyl acetate fraction (78.50 g, 60.73%), *n-*butanol fraction (17.18 g, 13.21%) and aqueous fraction (16.73 g, 12.76%). All extracts were stored in the refrigerator before the use.

### 2.3. Fingerprint Analysis by HPLC

HPLC was performed with a Hitachi Liquid Chromatography (Hitachi Ltd., Tokyo, Japan), consisting of two model L-5000 pumps, and one model L-7455 photodiode array detector (254 nm). Samples (10 mg/mL) were filtered through a 0.45 *μ*m PVDF-filter and injected into the HPLC column. The injection volume was 10 *μ*L and the separation temperature was 40°C. The column was a Mightysil RP-18 GP (5 *μ*m, 250 mm × 4.6 mm I.D.). The method involved the use of a binary gradient with mobile phases containing: (A) phosphoric acid in water (0.6‰, *v/v*) and (B) MeOH (*v/v*). The solvent gradient elution program was as follows: from 88% A to 78% A in 30 min, from 78% A to 68% A in 15 min. The flow rate was kept constant at 1.0 mL/min. A precolumn of *μ*-Bondapak C_18_ (Millipore, Milford, MA, USA) was attached to protect the analytical column. For photodiode array detection, the wavelengths of phenolic compounds at their respective maximum absorbance wavelength can be monitored at the same time. Identification is based on retention times and on-line spectral data in comparison with authentic standards. Quantification is performed by establishing calibration curves for each determined compound, using the standards.

### 2.4. *In Vitro* Antioxidant Activities of Crude Extracts

#### 2.4.1. Determination of Antioxidant Activity by DPPH Radical Scavenging Ability

The effects of crude extracts and positive controls (BHT) on DPPH radicals were estimated according to the method of Huang et al. [11]. Aliquot (20 *μ*L) of crude extracts at various concentrations were each mixed with 100 mM Tris-HCl buffer (80 *μ*L, pH 7.4) and then with 100 *μ*L of DPPH in ethanol to a final concentration of 250 *μ*M. The mixture was shaken vigorously and left to stand at room temperature for 20 min in the dark. The absorbance of the reaction solution was measured spectrophotometrically at 517 nm. The percentages of DPPH decolorization of the samples were calculated according to the following equation: % decolorization = [1 − (ABS_sample_/ABS_control_)] × 100. EC_50_ value was the effective concentration at which DPPH radicals were scavenged by 50% and was obtained by interpolation from linear regression analysis.

#### 2.4.2. Determination of Antioxidant Activity by ABTS^*·*+^ Scavenging Ability

The ABTS^*·*+^ scavenging ability was determined according to the method of [12]. Aqueous solution of ABTS (7 mM) was oxidized with potassium peroxodisulfate (2.45 mM) for 16 hrs in the dark at room temperature. The ABTS^*·*+^ solution was diluted with 95% ethanol to an absorbance of 0.75 ± 0.05 at 734 nm (Beckman UV-Vis spectrophotometer, Model DU640B). An aliquot (20 *μ*L) of each sample was mixed with 180 *μ*L ABTS^*·*+^ solution and the absorbance was read at 734 nm after 1 min. Trolox was used as a reference standard. 

#### 2.4.3. Determination of Total Polyphenol Content

The total polyphenol contents of crude extracts were determined according to the method of [12]. 20 *μ*L of each extract was added to 200 *μ*L distilled water and 40 *μ*L of Folin-Ciocalteu reagent. The mixture was allowed to stand at room temperature for 5 min and then 40 *μ*L of 20% sodium carbonate was added to the mixture. The resulting blue complex was then measured at 680 nm. Catechin was used as a standard for the calibration curve. The polyphenol content was calibrated using the linear equation based on the calibration curve. The total polyphenol content was expressed as mg catechin equivalence (CE)/g dry weight. 

#### 2.4.4. Determination of Total Flavonoid Content

The flavonoid content was determined according to the method of Lamaison and Carnat (1990) [13]. 100 *μ*L aliquots of the extract and fractions were added to equal volumes of 2% AlCl_3_·6H_2_O solutions. The mixtures were shaken vigorously and left incubating for 10 minutes before the absorbance was read at 430 nm. Rutin was used as standard for the calibration curve, by which a linear equation was derived to determine total flavonoid contents of the samples. Total flavonoid data were expressed in mg of rutin equivalents (RE)/g dry weight.

### 2.5. Cell Culture

A murine macrophage cell line RAW 264.7 (BCRC no. 60001) was purchased from the Bioresources Collection and Research Center (BCRC) of the Food Industry Research and Development Institute (Hsinchu, Taiwan). Cells were cultured in plastic dishes containing Dulbecco's Modified Eagle Medium (DMEM, Sigma, St. Louis, MO, USA) supplemented with 10% fetal bovine serum (FBS, Sigma, USA) in a CO_2_ incubator (5% CO_2_ in air) at 37°C and subcultured every 3 days at a dilution of 1 : 5 using 0.05% trypsin-0.02% EDTA in Ca^2+^-, Mg^2+^-free phosphate-buffered saline (DPBS). 

#### 2.5.1. Cell Viability

Cells (2 × 10^5^) were cultured in 96-well plates containing DMEM supplemented with 10% FBS for 1 day to become nearly confluent. Then cells were cultured with samples in the presence of 100 ng/mL LPS for 24 hrs. After that, the cells were washed twice with DPBS and incubated with 100 *μ*L of 0.5 mg/mL MTT for 2 hrs at 37°C testing for cell viability. The medium was then discarded and 100 *μ*L dimethyl sulfoxide (DMSO) was added. After 30 min incubation, absorbance at 570 nm was read by using a microplate reader.

#### 2.5.2. Measurement of Nitric Oxide/Nitrite

Nitrite levels in the cultured media and serum, which reflect intracellular NO synthase activity, were determined by Griess reaction [12]. The cells were incubated with samples in the presence of LPS (100 ng/mL) at 37°C for 24 hrs. Then, cells were dispensed into 96-well plates, and 100 *μ*L of each supernatant was mixed with the same volume of Griess reagent (1% sulfanilamide, 0.1% naphthyl ethylenediamine dihydrochloride and 5% phosphoric acid) and incubated at room temperature for 10 min. By using sodium nitrite to generate a standard curve, the concentration of nitrite was measured form absorbance at 540 nm. 

### 2.6. Animals

This study was conducted in conformity with the policies and procedure details in the “Guide for the Care and Use of Laboratory Animals” (NIH Publication no. 86–23 1985) and was approved by the ethics committee of the Institutional Animal Care and Use Committee (IACUC) of China Medical University, Taichung, Taiwan. ICR strain male mice (6–8 weeks old) were obtained from BioLASCO Taiwan Co., Ltd., Taipei, Taiwan. The animals were housed in an environmentally controlled room (temperature 22 ± 1°C; relative humidity 55 ± 5%; 12 h dark-light cycle). They were given food and water *ad libitum*.

After a 2-week adaptation period, male ICR mice (18–25 g) were randomly assigned to five groups (*n* = 6) of the animals in acetic acid-induced writhing (1%, 0.1 mL/10 g *i.p.*) and formalin-induced licking (5%, 20 *μ*L/per mice *i.p.*) experiments. These include a pathological model group (received acetic acid or formalin), a positive control (acetic acid or formalin + Indo), and EAAC administered groups (acetic acid or formalin + EAAC: 62.5, 125, and 250 mg/Kg). In the Carr-induced edema experiment, they were randomly assigned to six groups (*n* = 6) of the animals in the study. The control group receives normal saline (*i.p.*). The other five groups include a Carr-treated, a positive control (Carr + Indo), andEAAC administered groups (Carr + EAAC: 62.5, 125, and 250 mg/Kg).

#### 2.6.1. Acetic Acid-Induced Writhing Response

After a 2-week adaptation period, male ICR mice (18 to 25 g) were randomly assigned to six groups (*n* = 8) including a normal control, an Indo positive control, and four EAAC-treated groups. Control group received 1% acetic acid (10 mL/Kg body weight) and the positive control group received Indo (10 mg/Kg,* i.p.*) 25 min before intraperitoneal injection of 1% acetic acid (10 mL/Kg body weight). EAAC-treated groups received EAAC (62.5, 125, and 250 mg/Kg, *p.o.*) 55 min before intraperitoneal injection of 1% acetic acid (10 mL/Kg body weight). Five minutes after the *i.p*. injection of acetic acid, the number of writhing during the following 10 minutes was recorded [2]. 

#### 2.6.2. Formalin Test

The antinociceptive activity of the drugs was determined using the formalin test [3]. Control group received 5% formalin. Twenty microliters of 5% formalin were injected into the dorsal surface of the right-hind paw 60 min after administration of EAAC (62.5, 125, and 250 mg/Kg, *p.o.*) and 30 min after administration of Indo (10 mg/Kg, *i.p.*). The mice were observed for 30 min after the injection of formalin, and the amount of time took licking the injected hind paw was recorded. The first 5 min post formalin injection is referred to as the early phase and the period between 15 min and 40 min as the late phase. The total time took licking or biting the injured paw (pain behavior) was measured with a stop watch. The activity was recorded in 5 min intervals.

#### 2.6.3. Determination of Carrageenan (Carr) Induced Edema

Carr-induced hind paw edema model was used for determination of anti-inflammatory activity [14]. After a 2-week adaptation period, male ICR mice (18 to 25 g) were randomly assigned to five groups (*n* = 6) including Carr, positive Indo control, and three EAAC-treated groups. Carr group received 1% Carr (50 *μ*L). EAAC at doses of 62.5, 125, and 250 mg/Kg was orally administered 2 hrs before the injection with 1% Carr (50 *μ*L) in the plantar side of right-hind paws of the mice. And Indo (10 mg/Kg) was intraperitoneally administered 90 min before the injection with 1% Carr (50 *μ*L) in the plantar side of right-hind paws of the mice. Paw volume was measured immediately after Carr injection at 1, 2, 3, 4, and 5 h intervals using a plethysmometer (model 7159, Ugo Basile, Varese, Italy). The degree of swelling induced was evaluated by *a* minus *b*, where *a*  was the volume of the right-hind paw after Carr treatment and *b* was the volume of the right-hind paw before Carr treatment. Indo was used as a positive control.

In the later experiment, the right-hind paw tissue was taken after 5 h. The right-hind paw tissue was rinsed in ice-cold normal saline, and immediately placed in cold normal saline four times its volume and homogenized at 4°C. Then the homogenate was centrifuged at 12,000 ×g for 5 min. The supernatant was obtained and stored at −20°C refrigerator for MDA and the antioxidant enzymes (CAT, SOD, and GPx) activities assays.

#### 2.6.4. Determination of Tissue Lipid Peroxidation

MDA was evaluated by the thiobarbituric acid reacting substances (TRARS) method [15]. Briefly, MDA reacted with thiobarbituric acid in the acidic high temperature and formed a red-complex TBARS. The absorbance of TBARS was determined at 532 nm.

#### 2.6.5. Measurement of Tumor Necrosis Factor (TNF-*α*)

The levels of TNF-*α* were determined using a commercially available ELISA kit (BioSource International, Inc., Camarillo, CA, USA) according to the instructions of the manufacturer. TNF-*α* was determined from a standard curve. 

#### 2.6.6. Determination of Antioxidant Enzyme Activity in Paw Tissue

The following biochemical parameters were analyzed to check the protective activity of EAAC by the methods given below. Total SOD activity was determined by the inhibition of cytochrome *c* reduction [16]. The reduction of cytochrome *c* was mediated by superoxide anions generated by the xanthine/xanthine oxidase system and monitored at 550 nm. One unit of SOD was defined as the amount of enzyme required to inhibit the rate of cytochrome *c* reduction by 50%. Total CAT activity estimation was based on the previously reported [17]. In brief, the reduction of 10 mM H_2_O_2_ 20 mM of phosphate buffer (pH 7) was monitored by measuring the absorbance at 240 nm. The activity was calculated by using a molar absorption coefficient, and the enzyme activity was defined as nanomoles of dissipating hydrogen peroxide per milligram protein per minute. Total GPx activity in cytosol was determined as previously reported [18]. The enzyme solution was added to a mixture containing hydrogen peroxide and glutathione in 0.1 mM Tris buffer (pH 7.2) and the absorbance at 340 nm was measured. Activity was evaluated from a calibration curve, and the enzyme activity was defined as nanomoles of NADPH oxidized per milligram protein per minute. The protein concentration of the tissue was determined by the Bradford dye-binding assay (Bio-Rad, Hercules, CA, USA).

#### 2.6.7. Protein Lysate Preparation and Western Blot Analysis of iNOS, COX-2, and HO-1

Total protein was extracted with RIPA solution (radioimmunoprecipitation assay buffer) at −20°C overnight. We used BSA (bovine serum albumin) as a protein standard to calculate equal total cellular protein amounts. Protein samples (30 *μ*g) were resolved by denaturing sodium dodecyl sulfate-polyacrylamide gel electrophoresis (SDS-PAGE) using standard methods, and then were transferred to PVDF membranes by electroblotting and blocking with 1% BSA. The membranes were probed with the primary antibodies (iNOS, COX-2, HO-1, and *β*-actin) at 4°C overnight, washed three times with PBST, and incubated for 1 h at 37°C with horseradish peroxidase conjugated secondary antibodies. The membranes were washed three times and the immunoreactive proteins were detected by enhanced chemiluminescence (ECL) using hyperfilm and ECL reagent (Amersham International plc., Buckinghamshire, UK). The results of western blot analysis were quantified by measuring the relative intensity compared to the control using Kodak Molecular Imaging Software and represented in the relative intensities.

#### 2.6.8. Histological Examination

For histological examination, biopsies of paws were taken 5th hrs following the interplanetary injection of Carr. The tissue slices were fixed in (1.85% formaldehyde, 1% acetic acid) for 1 week at room temperature, dehydrated by graded ethanol, and embedded in paraffin (Sherwood Medical). Sections (thickness 5 *μ*m) were deparaffinized with xylene and stained with H&E stain. All samples were observed and photographed with BH2 Olympus microscopy. Every 3~5 tissue slices were randomly chosen from Carr, Indo, and EAAC treated (250 mg/Kg) groups. The numbers of neutrophils were counted in each scope (400x) and thereafter we obtained their average count from 5 scopes of every tissue slice [6].

### 2.7. Statistical Analysis

Experimental results were presented as the mean ± standard deviation (SD) of three parallel measurements. IC_50_ values were estimated using a nonlinear regression algorithm (SigmaPlot 8.0; SPSS Inc., Chicago, IL, USA). Data obtained from animal experiments were expressed as mean standard error (± S.E.M.). Statistical evaluation was carried out by one-way analysis of variance (ANOVA followed by Scheffe's multiple range tests). Statistical significance is expressed as *P < 0.05, **P < 0.01, and ***P < 0.001.

## 3. Results

### 3.1. Fingerprint Analysis by HPLC

**Figure 1:**
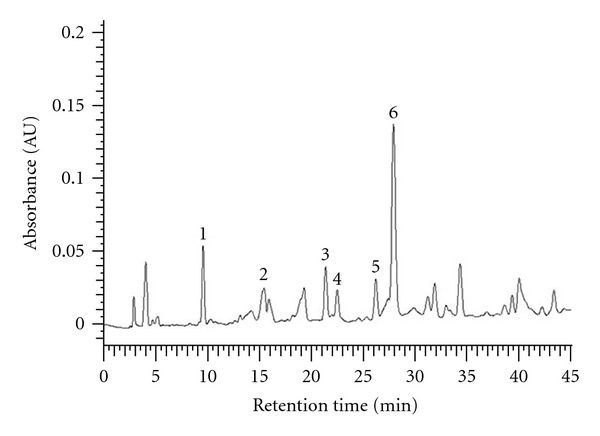
HPLC chromatograms (280 nm) of ethyl-acetate fraction of the stem of* A. callosa *var. *callosa*. 1: protocatechuic acid; 2: catechin; 3: vanillic acid; 4: caffeic acid; 5: syringic acid; and 6: epicatechin.

To establish the fingerprint chromatogram for the quality control of EAAC, protocatechuic acid, catechin, vanillic acid, caffeic acid, syringic acid, and epicatechin were used as markers. An optimized HPLC-PAD technique was employed. Meanwhile, HPLC chromatograms showed five marker components present in EAAC. As shown in Figure 1, these phenolic components have been identified as protocatechuic acid, catechin, vanillic acid, caffeic acid, syringic acid, and epicatechin by their retention time and UV absorbance of purified standards. According to the plot of peak-area ratio (*y*) versus concentration (*x*, *μ*g/mL), the regression equations of the five constituents and their correlation coefficients (*r*) were determined as follows: protocatechuic acid, *y* = 0.622*x* + 4.301  (*r* ^2^ = 0.999); catechin, *y* = 0.443*x* + 3.104  (*r* ^2^ = 0.999); vanillic acid, *y* = 0.836*x* + 5.686  (*r* ^2^ = 0.999); caffeic acid, *y* = 1.643*x* + 11.65  (*r* ^2^ = 0.999), syringic acid, *y* = 1.410*x* + 10.19  (*r* ^2^ = 0.999); epicatechin, *y* = 0.622*x* + 4.30  (*r* ^2^ = 0.999). The relative amounts of the six phenolic compounds found in EAAC were in the order of epicatechin (35.09 mg/g extract) > catechin (29.22 mg/g extract) > protocatechuic acid (21.84 mg/g extract) > caffeic acid (16.73 mg/g extract) > vanillic acid (14.28 mg/g extract) > and syringic acid (5.71 mg/g extract), respectively.

### 3.2. Trolox Equivalent Antioxidant Capacity (TEAC)

 TEAC assay is often used to evaluate the total antioxidant power of single compounds and complex mixtures of various plants.  Table 1 shows TEAC values of the methanol extract of *A. callosa *var. *callosa* (MAC) and fractions. TEAC value of the MAC extract was 89.87 ± 1.58 *μ*g/mg extract. As for the fractions, EA fraction of *A. callosa *var. *callosa* (EAAC) exhibited the strongest antioxidant activity (130.69 ± 2.17 *μ*g/mg extract), followed by *n*-butanol fraction (125.86 ± 1.47 *μ*g/mg extract), water fraction (98.43 ± 1.05 *μ*g/mg extract), and *n-*hexane fraction (14.26 ± 0.59 *μ*g/mg extract). As shown in Table 1, reference compounds protocatechuic acid, catechin, vanillic acid, caffeic acid, syringic acid, and epicatechin in the EAAC showed TEAC value of 1852.14 ± 2.87, 2013.36 ± 4.53, 832.75 ± 4.38, 2137.28 ± 6.32, 1929.10 ± 5.37, and 1657.56 ± 3.59 *μ*g/mg extract, respectively.

#### 3.2.1. Scavenging Activity against 1, 1-Diphenyl-2-Picrylhydrazyl Radical

The relatively stable organic radical DPPH is widely used in modeling systems to investigate the scavenging activities of several natural compounds, such as phenolics and anthocyanins, as well as crude extract of plants [3]. EAAC exhibited the strongest antioxidant activities in scavenging DPPH radicals, with IC_50_ values of 132.69 ± 4.32 mg/mL, respectively (Table 1). As shown in Table 1, reference compounds protocatechuic acid, catechin, vanillic acid, caffeic acid, syringic acid, and epicatechin in the EAAC showed DPPH radical scavenging with an EC_50_ value of 12.46 ± 1.57, 9.45 ± 0.54, 46.84 ± 2.62, 8.57 ± 0.54, 10.25 ± 0.35, and 20.72 ± 1.23 *μ*g/mL, respectively.

#### 3.2.2. Determination of Total Phenolic and Total Flavonoid Contents in the Plant Extract and Fractions

Total phenolic content was expressed as mg of catechin equivalent per gram of dry weight. The results showed that EAAC had the highest phenolic contents of 519.54 ± 2.47 mg CE/g, respectively (Table 1). Total flavonoid content was expressed as mg of rutin equivalent per gram of dry weight. The results revealed that the total flavonoid contents of the extract and fractions varied from 18.67 to 6.84 mg RE/g. Among all the fractions, EAAC had the highest total flavonoid content of 18.67 ± 0.49 mg RE/g.

### 3.3. Effect of the MAC Extract and Fractions on LPS-Induced NO Production in Macrophages

**Figure 2:**
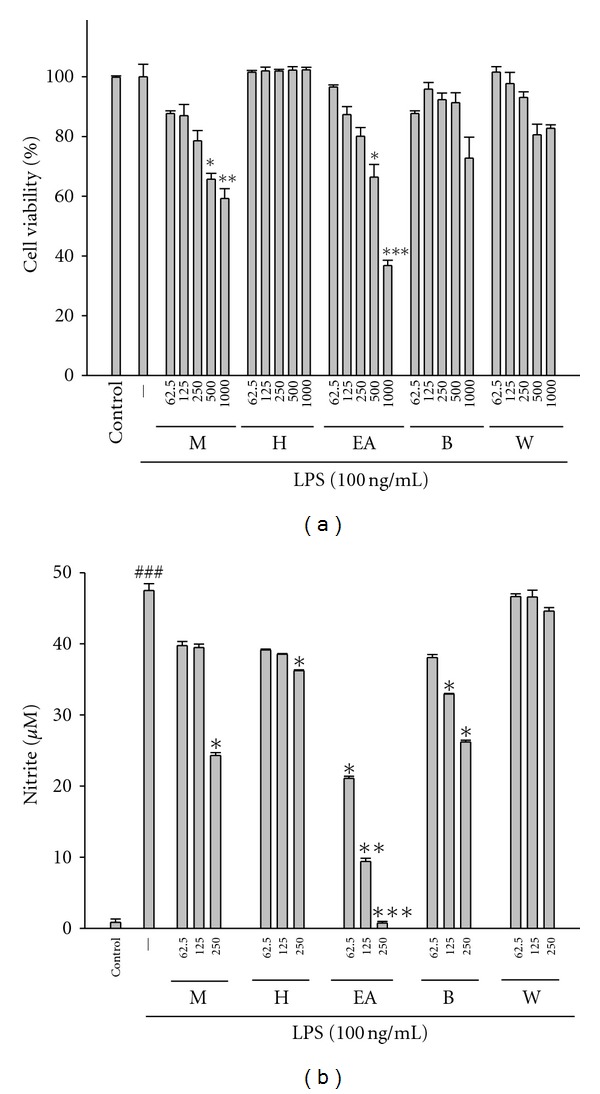
The effects of the methanolic extract and fractions from the stem of* A. callosa *var. *callosa *on lipopolysaccharide-(LPS-)induced cell viability (a) and NO production (b) of RAW 264.7 macrophages. Cells were incubated for 24 h with 100 ng/mL of LPS in the absence or presence of samples (0, 125, 250, 500, and 1000 *μ*g/mL). Cell viability assay was performed using MTT assay. Nitrite concentration in the medium was determined using Griess reagent. M: methanol extract; H: *n*-hexane fraction; EA: ethyl-acetate fraction; B: *n*-butanol fraction; W: water fraction; The data were presented as mean ± S.D. for three different experiments performed in triplicate. ^###^Compared with sample of control group. *P < 0.05, **P < 0.01, and ***P < 0.001 were compared with LPS-alone group.

The effect of the MAC extract and fractions on RAW264.7 cell viability was determined by an MTT assay. Cells cultured with the MAC extract and fractions at the concentrations (0, 62.5, 125, and 250 *μ*g/mL) used in the presence of 100 ng/mL LPS for 24 h did not change cell viability, significantly (Figure 2(a)). In the present study, effects of the MAC extract, its fractions, and reference compounds on LPS-induced NO production in RAW 264.7 macrophages were investigated. Nitrite accumulated in the culture medium was estimated by the Griess reaction as an index for NO release from the cells. After treatment with LPS (100 ng/mL) for 24 h, the nitrite concentration increased in the medium. The MAC extract and fractions did not interfere with the reaction between nitrite and Griess reagents at 250 *μ*g/mL (data not shown). Unstimulated macrophages, after 24 h of incubation in culture medium produced background levels of nitrite. When RAW264.7 macrophages were treated with different concentrations of the MAC extract and fractions together with LPS for 24 h, the MAC extract and fractions inhibited nitrite production significantly (Figure 2(b)). When RAW264.7 macrophages were treated with different concentrations of EAAC (0, 62.5, 125, and 250 *μ*g/mL) together with LPS for 24 h, a significant concentration-dependent inhibition of nitrite production was detected. And the reference compounds of catechin and caffeic acid in the EAAC also showed the NO inhibitory activity induced by LPS in RAW264.7 macrophages with an IC_50_ value of 86.18 ± 0.15 and 32.26 ± 0.23 *μ*g/mL, respectively. Protocatechuic acid, vanillic acid, syringic acid, and epicatechin had weakened the NO inhibitory activity induced by LPS in RAW264.7 macrophages, respectively. Hence, we evaluated the reference compounds in the EAAC that catechin and caffeic acid were studieo on the pharmacological activities by free radical scavenging and LPS-induced NO production in RAW264.7 macrophages.

### 3.4. Inhibition of LPS-Induced Level of TNF-*α* by EAAC

**Figure 3:**
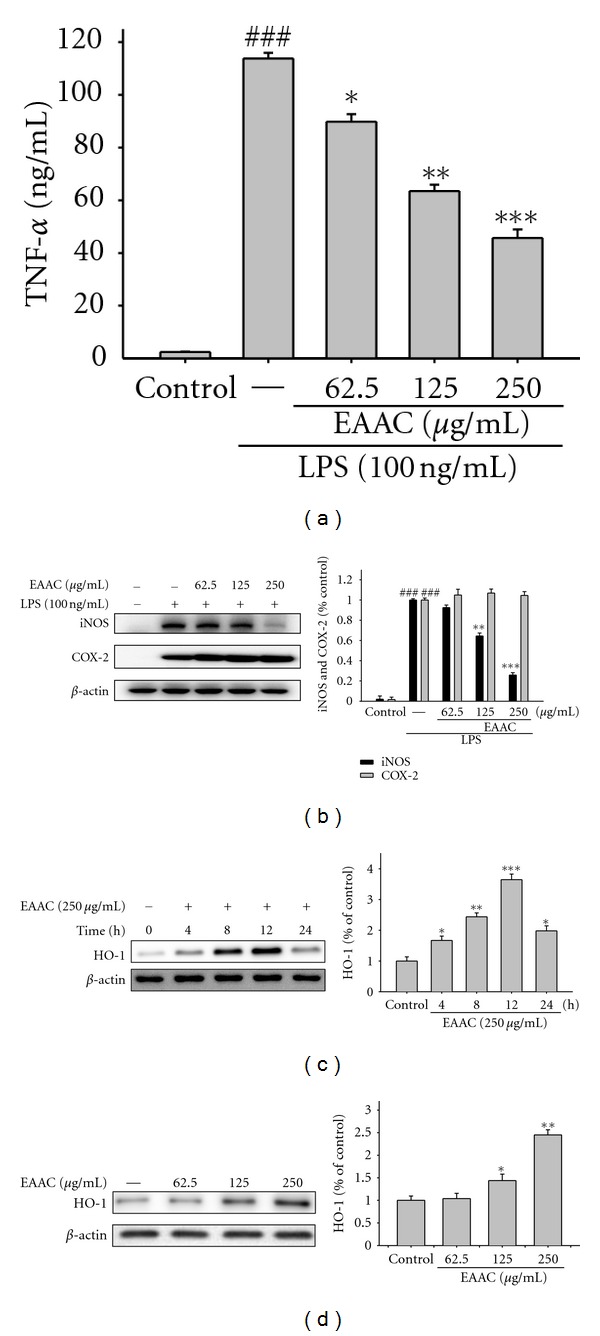
The effects of ethyl-acetate fractions from the stem of *A. callosa* var. *callosa* (EAAC) on lipopolysaccharide-(LPS-) induced TNF-*α* (a), iNOS, COX-2 (b), and HO-1 ((c) and (d)) expressions. TNF-*α* concentration in the medium was determined using ELISA kit. The protein expression of iNOS and COX-2 by cells were incubated for 24 h with 100 ng/mL of LPS in the absence or the presence of EAAC (0, 62.5, 125, and 250 *μ*g/mL). The protein expression of HO-1 by cells were incubated for the indicated times or different concentration of EAAC (0, 62.5, 125, and 250 *μ*g/mL) for 12 h. Lysed cells were then prepared and subjected to western blotting using an antibody specific for iNOS, COX-2, and HO-1. *β*-actin was used as an internal control. Representative western blot from two separate experiments is shown. Relative iNOS, COX-2, and HO-1 protein levels were calculated with reference to a LPS-stimulated culture. ^###^Compared with sample of control group. The data were presented as mean ± S.D. for three different experiments performed in triplicate. *P < 0.05, **P < 0.01, and ***P < 0.001 were compared with LPS-alone group.

We examined the effect of EAAC on LPS-induced upregulation of TNF-*α*. A very low amount of TNF-*α* protein was detected by a specific ELISA for TNF-*α* in controls (Figure 3(a)). When RAW264.7 macrophages were treated with different concentrations of EAAC (0, 62.5, 125, and 250 *μ*g/mL) together with LPS for 24 h, a significant concentration-dependent inhibition of TNF-*α* production was detected. There was either a significant decrease in the TNF-*α* production of group treated with 62.5 *μ*g/mL EAAC (*P* < 0.05), or highly significant decrease of groups treated, respectively, with 125 and 250 *μ*g/mL of EAAC when compared with the LPS-alone group (*P* < 0.01 or *P* < 0.001). The IC_50_ value for inhibition of TNF-*α* production of EAAC was about 189.32 ± 2.14 *μ*g/mL. 

#### 3.4.1. Inhibition of LPS-Induced iNOS and COX-2 Protein by EAAC

The results showed that incubation with EAAC in the presence of LPS for 24 h inhibited iNOS protein expression but not COX-2 protein in mouse macrophage RAW264.7 cells in a dose-dependent manner (Figure 3(b)). The intensity of protein bands was analyzed and showed an average of 74.3% downregulation of iNOS proteins, respectively, after treatment with EA fraction at the 250 *μ*g/mL compared with the LPS-alone group.

#### 3.4.2. EAAC Induces HO-1 Expression in Macrophage Cells

RAW 264.7 cells were treated with EAAC at various concentrations and for various times of exposure to determine the potential effects on HO-1 expression. As shown in Figure 3(b), HO-1 protein levels were induced after the indicated periods of time and peaked after 12 h of EAAC treatment in RAW 264.7 cells. In agreement with what we observed with HO-1, protein levels increased following EAAC treatment in a dose-dependent manner (Figure 3(d)).

### 3.5. Acetic Acid-Induced Writhing Response

**Figure 4:**
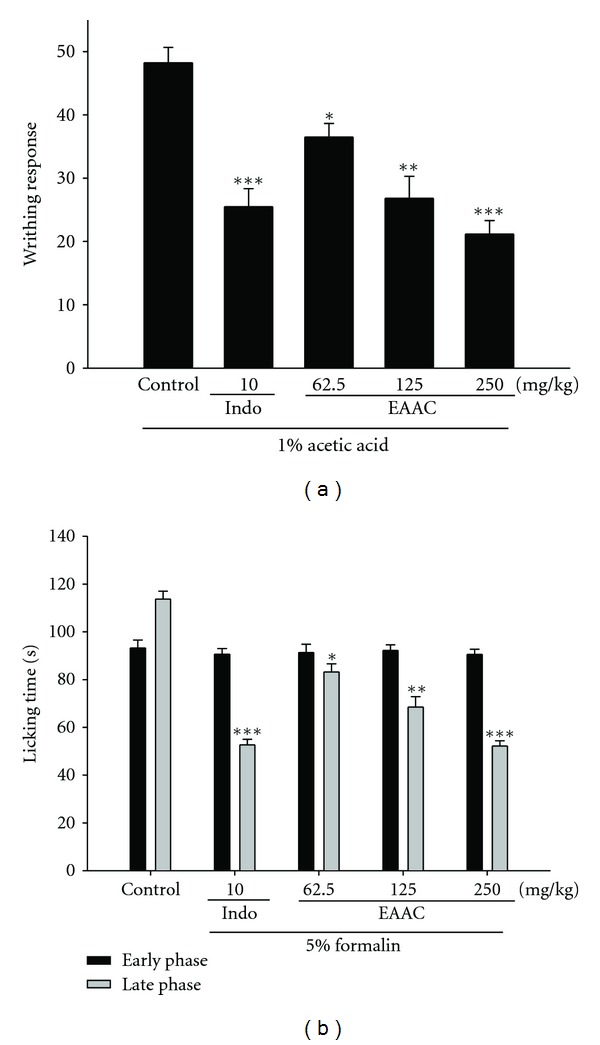
Analgesic effects of EAAC and indomethacin (Indo) on acetic acid-induced writhing response (a) and the early phase and late phase in formalin test (b) in mice. Each value represents mean ± S.E.M. *P < 0.05, **P < 0.01, and ***P < 0.001 as compared with the pathological model group (Con) (one-way ANOVA followed by Scheffe's multiple range test).

The cumulative amount of abdominal stretching correlated with the level of acetic acid-induced pain (Figure 4(a)). EAAC treatment (0, 62.5, 125, and 250 mg/Kg) significantly inhibited the number of writhing in comparison with the pathological model group. The inhibition rates of the number of writhing compared with the pathological model group are 24.27%, 44.38%, and 56.14%, respectively. The inhibiting effect of acetic acid-induced writhing by EAAC (250 mg/kg) was similar to that produced by a positive control Indo (10 mg/kg) (*P* < 0.001). 

#### 3.5.1. Formalin Test

EAAC significantly inhibited formalin-induced pain in the late phase; however, there was no inhibition in the early phase (Figure 4(b)). EAAC treatment (0, 62.5, 125, and 250 mg/Kg) significantly inhibited the formalin-induced pain (late phase) in comparison with the pathological model group. The inhibition rates of formalin-induced licking compared with the pathological model group are 26.86%, 39.75%, and 53.83%, respectively. This inhibiting effect of formalin-induced licking time by EAAC (250 mg/kg; *P* < 0.001) was better than a positive control Indo (10 mg/kg) (*P* < 0.001).

#### 3.5.2. *λ*-Carrageenan-(Carr-)Induced Edema

**Figure 5:**
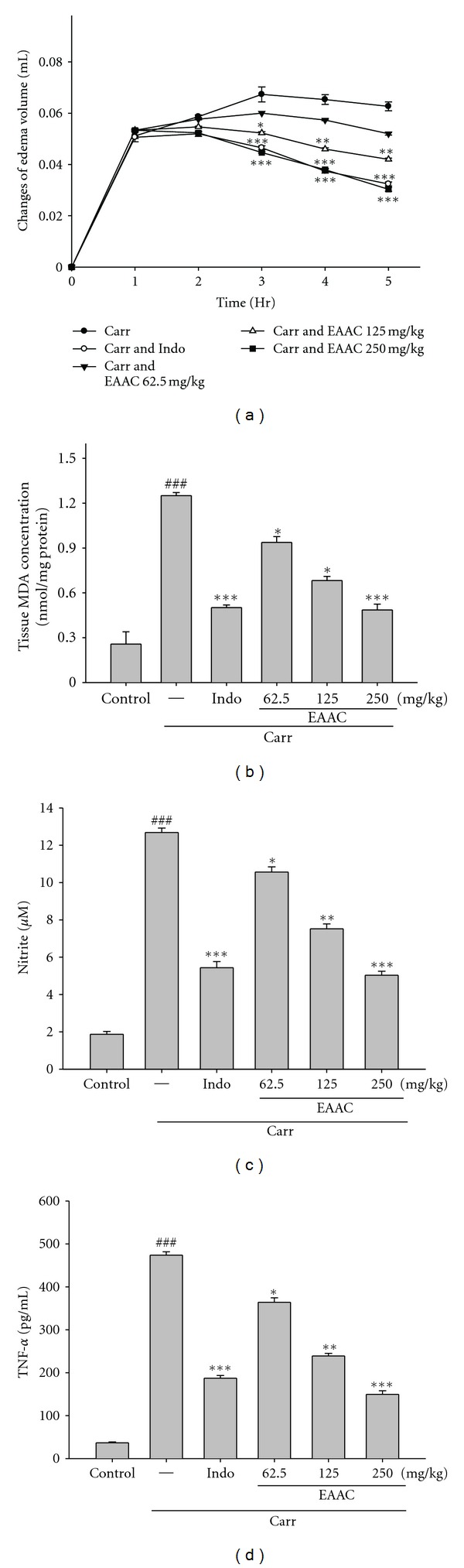
Effects of EAAC and Indo on hind paw edema induced by Carr in mice (a), the tissue MDA concentration of foot in mice (b), Carr-induced NO (c), and TNF-*α* (d) concentrations of serum at the 5th hour in mice. Each value represents as mean ± S.E.M. *P < 0.05, **P < 0.01, and ***P < 0.001 as compared with the Carr group (one-way ANOVA followed by Scheffe's multiple range test).

Figure 5(a) shows the effect of EAAC on Carr induced paw edema in mice. Indo is an anti-inflammatory drug used to reduce acute inflammatory response such as swelling. According to Figure 5(a), Indo (10 mg/Kg) reduced the edema volumes about 48.32% in comparison to the Carr group during the 5^th^ h of Carr treatment. Further, in the range of 62.5–250 mg/Kg, EAAC showed a concentration-dependent inhibition of edema development. For EAAC at the concentration of 250 mg/Kg, the levels of edema volume were decreased to 51.67% of that observed in the Carr group after 5th hour treatment. These data imply that EAAC can exhibit an inhibitor of edema in acute inflammatory processes. 

#### 3.5.3. Effects of EAAC on MDA, NO, and TNF-*α* Levels

Lipid oxidation serves as a marker of cellular damage and has been recognized as a marker of inflammatory damage. As shown in Figure 5(b), Carr increased the level of lipid oxidation by 4.86-folds in comparison with the control group. Meanwhile, Indo decreased the level of lipid oxidation to 60.02% of that observed in the Carr group. In fact, in the range of 62.5–250 mg/Kg, EAAC inhibited the level of lipid oxidation down to 25.63–61.15% of that observed in the Carr group. These data imply that EAAC can protect against tissue lipid oxidation in Carr-induced inflammatory processes.

Carr-induced inflammatory processes increased NO and elevated TNF-*α* production. As shown in Figure 5(c), Carr increased the level of nitrite by 6.79-folds in comparison to the control group in serum. Meanwhile, Indo decreased the level of serum nitrite to 57.13% of that observed in the Carr group. In fact, in the range of 62.5–250 mg/Kg, EAAC reduced the level of nitrite to 16.71–60.33% of that observed in the Carr group. And Carr increased the level of TNF-*α* in the serum by 8.34-folds in comparison to the control group (Figure 5(d)). Indo decreased the level of serum TNF-*α* to 60.46% of that observed in the Carr group. EAAC also inhibited the production of TNF-*α* to 23.16–68.54% of that observed in the Carr group. These data-provide evidence that EAAC acts as an inhibitor of Carr-induced tissue inflammation by decreasing NO and TNF-*α* productions *in vivo*.

#### 3.5.4. Effects of EAAC on the Activities of Antioxidant Enzymes in Carr-Induced Paw Edema

Table 2 shows the activities of CAT, SOD, and GPx in Carr-induced paw edema of treated mice. Carr decreased the activities of CAT, SOD, and GPx in Carr-induced paw edema by 36.63%, 43.45%, and 37.39.3%, respectively, in comparison to the control group (*P* < 0.001). In the range of 62.5–250 mg/Kg, EAAC increased the activities of CAT to 104.13%–149.85%, SOD to 115.04%–164.13%, and GPx to 114.86%–143.76%, respectively, compared to that observed in the Carr group. Indo also exhibited increase effects in the activities of CAT (143.06%), SOD (161.79%), and GPx (137.21%) in comparison to the Carr group. These data imply that the anti-inflammatory effects of EAAC *in vivo* might be attributed to its elevation in the antioxidant enzymes activities of Carr-induced mice.

#### 3.5.5. Effects of EAAC on Carr-Induced iNOS, COX-2, and HO-1 Protein Expressions in Mice Paw Edema

**Figure 6:**
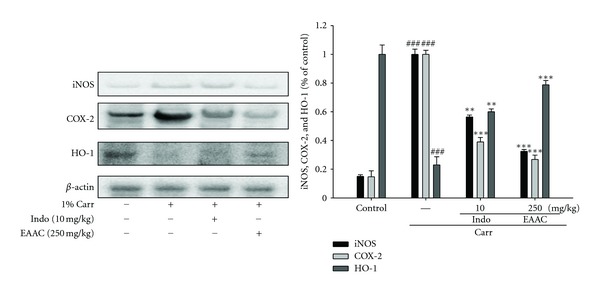
The protein expressions of iNOS, COX-2, and HO-1 by EAAC induced by Carr of foot at the 5th hour in mice. Tissue suspended was then prepared and subjected to western blotting using an antibody specific for iNOS, COX-2, and HO-1. *β*-actin was used as an internal control. A representative western blot from two separate experiments is shown. Relative iNOS, COX-2, and HO-1 protein levels were calculated with reference to a Carr-injected mouse. ^###^Compared with sample of control group. The data were presented as mean ± S.D. for three different experiments performed in triplicate. **P < 0.01 and ***P < 0.001 were compared with Carr-alone group.

The results showed that administration of EAAC (250 mg/Kg) on Carr-induced for 5th hour inhibited iNOS and COX-2 proteins expression in mouse paw edema (Figure 6). The intensity of protein bands was analyzed and showed an average of 77.58% and 73.21% down-regulation of iNOS and COX-2 protein (*P* < 0.001) respectively, after treatment with EAAC at 250 mg/Kg compared with the Carr-induced alone (Figure 6). In addition, the protein expression showed an average of 43.65% and 61.23% downregulation of iNOS and COX-2 protein after treatment with Indo at 10 mg/Kg compared with the Carr-induced alone. And we also found that the intensity of protein bands showed an average of 78.8% up-regulation of HO-1 protein (*P* < 0.001), (Figure 6).

#### 3.5.6. Histological Examination

**Figure 7:**
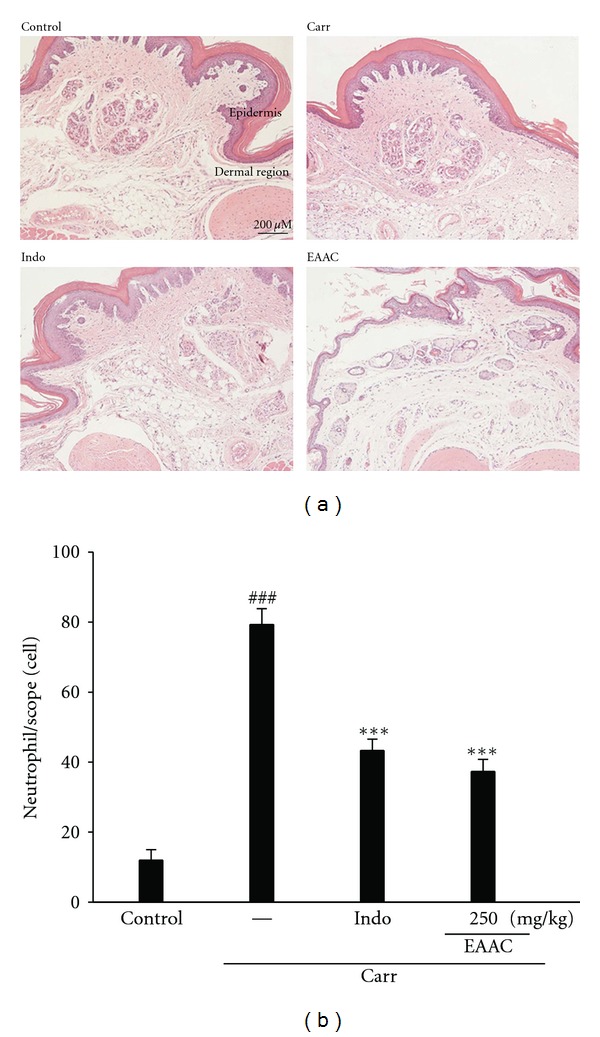
Representative light micrographs of mouse hind footpad H&E stained to reveal hemorrhage, edemas and inflammatory cell infiltration in control mice (a), Carr-treated mice demonstrates hemorrhage with moderately extravascular red blood cell and large amount of inflammatory leukocyte mainly neutrophils infiltration in the subdermis interstitial tissue of mice, and mice given indomethacin (Indo) (10 mg/kg) before Carr. EAAC significantly shows morphological alterations (100x) and the numbers of neutrophils in each scope (400x) (b) compared to subcutaneous injection of Carr only. ^###^ *P* < 0.001 as compared with the control group. The data were presented as mean ± S.D. for three different experiments performed in triplicate. ***P < 0.001 compared with Carr group. Scale bar = 200 *μ*m.

The 5th hour peak of the Carr inflammatory response was found to be associated with subcutaneous edema along with the heavy infiltration of inflammatory cells, particularly neutrophils, at the site of injection as compared to the control. Further, paw Carr-induced biopsies of animals treated with EAAC showed a reduction in inflammatory response. Actually, inflammatory cells were reduced in number and were confined near the vascular areas. Intercellular spaces did not show any cellular infiltrations. Collagen fibers were regular in shape and showed a reduction of intercellular spaces. Moreover, the hypoderm connective tissue was not damaged (Figure 7(a)). Neutrophils were notified and increased with Carr treatment (*P* < 0.001). Indo and EAAC (250 mg/Kg) could significantly decrease the neutrophils numbers as compared to the Carr-treated group (*P* < 0.001) (Figure 7(b)).

## 4. Discussion

Polyphenols are secondary metabolism products of plants and constitute one of the most numerous and widely distributed groups of natural antioxidants in the plant. Polyphenols act as antioxidants via several mechanisms including the scavenging of free radicals and chelation of transition metals, as well as the mediation and inhibition of enzymes [19]. The use of traditional medicine is widespread and plants still present a large source of natural antioxidants that might serve as leads for the development of novel drugs. The higher radical scavenging activity of EAAC seems to be closely correlated with its polyphenolic constituents though active components could play important roles in its antioxidative effect. Consequently, it is possible that the total phenolic constituents may contribute to anti-inflammatory activity of EAAC. In this paper, we demonstrated that EAAC inhibited radical scavenging and NO production. And the reference compound of protocatechuic acid, catechin, vanillic acid, caffeic acid, syringic acid, and epicatechin in the EAAC also had the antioxidant activities (Table 1). 

Two different analgesic testing methods were employed with the objective of identifying possible peripheral and central effects of the test substances. The acetic writhing test is used to study the peripheral analgesic effects of drugs [20]. Related studies have demonstrated that acetic acid acts indirectly by inducing the release of endogenous mediators which stimulate the nociceptive neurons sensitive to nonsteroidal anti-inflammatory drugs (NSAIDs) [21]. When compared with antinociceptive activities, EAAC was relatively potent in acetic acid writhing test indicating peripheral antinociception. In contrast, EAAC (250 mg/Kg) exhibited an action in similar magnitude with Indo, a reference drug for peripheral anti-nociception (Figure 4(a)). Formalin-induced paw pain produced a distinct biphasic nociception, a first phase (lasting the first 5 min; early acute phase) corresponding to acute neurogenic pain, and a second phase (lasting from 15 to 30 min; late phase) corresponding to inflammatory pain responses [22]. Therefore, the test can be used to clarify the possible mechanism of an antinociceptive effect of a proposed analgesic. The inhibitory effect of EAAC on the nociceptive response in the late phase of the formalin test suggested that the antinociceptive effect of EAAC could be due to its peripheral action (Figure 4(b)). 

Carr-induced inflammation has been well established as a valid model to study free radical generation in paw tissue after inflammatory states. The molecular mechanism of the Carr-induced inflammation is well characterized, and these models of inflammation are standard models of screening for anti-inflammatory activity of various experimental compounds [23]. The early phase of Carr edema is related to the production of histamine, leukotrienes, and possibly cyclooxygenase products, while the delayed phase of the Carr-induced inflammatory response has been linked to neutrophil infiltration and the production of neutrophil-derived free radicals, such as hydrogen peroxide, superoxide, and OH radicals, as well as to the release of other neutrophil-derived mediators such as TNF-*α* [24]. However, a reduction in paw swelling size is a good index in determining the protective action of anti-inflammatory agents. According to Figure 5(a), EAAC (250 mg/Kg) inhibited the development of edema at the 5th hours after treatment. 

In the process of inflammation, the overproduction of NO could induce cell damage as well as inflammation. Our data imply that the inhibitory effects of EAAC on NO production could contribute to the decrease of oxidative stress and inflammation development in tissues. Free radicals play an important role in the Carr-induced acute inflammatory response [25]. NO produced by activated macrophages also plays an important mediator in the cytotoxic/cytostatic mechanism of nonspecific immunity. Therefore, EAAC decreased NO production *in vitro *(Table 1) and* in vivo* (Figure 2(b)), which could further lead to reduce the edema response in inflammation. 

During inflammatory processes, large amounts of the proinflammatory mediators, NO and PGE_2_, are generated by inducible iNOS and COX-2, respectively. INOS is induced by various stimuli, which include bacterial LPS, TNF-*α*, IL-1*β*, and interferon-*γ* [26]. However, COX-2 is induced by proinflammatory cytokines and growth factors. It is clear that COX-2 enhanced production of the prostaglandins that mediate inflammation, pain and, fever and is the target enzyme for the anti-inflammatory activity of NSAIDs. In this study, there is a significant decrease in iNOS and COX-2 activities with EAAC treatment (Figures 3(b) and 6). We assume that the suppression of NO production is probably due to the decreases of iNOS activities. Therefore, the inhibitory effect of EAAC on NO production could be contributed to its total polyphenols inhibition to iNOS protein expression. 

Recently, several studies have demonstrated that HO-1 plays a regulatory role in the inflammatory response by inhibiting production of proinflammatory cytokines [7], suggesting a potential therapeutic strategy for treating inflammatory diseases. HO-1 catabolizes the rate limiting step in the degradation of cellular heme and produces three products, including CO, biliverdin (which is rapidly converted to bilirubin), and free irons (Fe^2+^) [6]. The potential anti-inflammatory properties of HO-1 are not only mediated through the degradation of pro-inflammatory free-heme, but also through the production of the antioxidant, antiapoptotic, and anti-inflammatory by-products, CO and bilirubin [27]. Thus, upregulation of HO-1 expression and the increase in HO-1 activity are targets for the treatment of inflammatory diseases. In the present study, we demonstrated that EAAC induces HO-1 expression in murine macrophages and paw edema. Exposure of RAW 264.7 cells to up to 250 *μ*g/mL EAAC increased the expression of HO-1 protein in a dose-dependent manner.

 Lipid oxidation not only serves as a marker of tissue damage *in vivo* but also has been recognized to be the inducer of inflammatory processes. Inflammatory effect induced by Carr is associated with free radicals. Free radicals, prostaglandin, and NO will be released when administrating with Carr for 1–6 hrs [28]. Lipid peroxidation is a process in which free radicals attack lipids in cell membranes. MDA production is due to free radical attack on plasma membrane. Thus, inflammatory effect would result in the accumulation of MDA. In this study, EAAC not only exhibited radicals scavenging capacity but also decreased Carr induced lipid damage* in vivo*. 

Cellular antioxidants are known to change their redox state, and they can regulate oxidative processes involved in signal transduction and protein expression. ROS has been proposed to mediate tissue damage via a number of independent mechanisms including the inactivation of a variety of antioxidant enzymes. Given the importance of the oxidative status in the formation of edema, the anti-inflammatory effect exhibited by drug in this model might be related to its antioxidant properties [29]. The role of CAT is to decompose H_2_O_2_. Increased SOD activity can protect cells against threat of reactive free radicals. GPx is regarded as a crucial enzyme which catalyses the reduction of hydroperoxide [30]. In this study, there was a significant increase in CAT, SOD, and GPx activities with EAAC treatment. The anti-inflammatory effect of EAAC could be due to elevated intracellular antioxidant enzyme activities and decreased inflammatory stress in tissue. These data suggest that EAAC could serve as a natural antioxidant to protect cells against inflammatory damage. 

Studies have noted that plant materials which contain tannins, alkaloids, flavonoids, and phenolic acids bring out antinociceptive and anti-inflammatory effects on experimental animals [21]. Preliminary phytochemical experiments indicated the presence of phenolic acids and flavonoids, which may be responsible for the antinociceptive activity of EAAC. Studies have also demonstrated that phenolic acids such as protocatechuic acid [31], catechin, epicatechin [32], and caffeic acid [33] produced significant antinociceptive and anti-inflammatory activities. Hence, it was suggested that the antioxidant, analgesic, and anti-inflammatory activities of EAAC may be related to its phenolic content.

In conclusion, our data suggest that EAAC shows anti-inflammatory effects *in vitro *and *in vivo*. Anti-inflammatory mechanisms of EAAC might be correlated to the expression of iNOS, COX-2, and HO-1 in physiological systems. The antioxidant effects of EAAC can be due to increase in the activities of antioxidant enzymes and its effects on radicals scavenging. Therefore, we suggest that EAAC contain herbal antioxidants and exhibit analgesic, anti-inflammatory activity *in vivo*. 

## Figures and Tables

**Table 1:** Radical scavenging activity of methanol extract of *Actinidia callosa *var. *callosa* (MAC) determined by TEAC, DPPH assay, and inhibition of NO production in LPS-induced RAW 264.7 macrophages by MAC and its reference compounds.^∗^

Samples	TEAC (*μ*g/mg extract)	DPPH scavenging activity EC_50_ (*μ*g/mL)	Total phenolic (mg CE/g)^a^	Total flavonoid (mg RE/g)^b^	NO production IC_50 _value (*μ*g/mL)
Methanol extract	90 ± 1.58	215.48 ± 3.59	299.92 ± 2.13	9.34 ± 0.41	237.24 ± 3.79
*n*-hexane fraction	14.26 ± 0.59	1754.29 ± 8.71	177.33 ± 1.49	8.29 ± 0.35	>250
Ethyl-acetate fraction	130.69 ± 2.16	132.69 ± 4.32	519.54 ± 2.47	18.67 ± 0.49	59.23 ± 2.17
*n*-butanol fraction	125.86 ± 1.47	135.31 ± 3.19	415.06 ± 1.47	6.22 ± 0.23	>250
Water fraction	98.43 ± 1.05	162.78 ± 1.15	361.45 ± 1.29	6.84 ± 0.51	>250
Protocatechuic acid	1552.14 ± 2.87	12.46 ± 1.57	(—)	(—)	>100
Catechin	1613.36 ± 4.53	9.45 ± 0.54	(—)	(—)	86.18 ± 0.15
Vanillic acid	532.75 ± 4.38	46.84 ± 2.62	(—)	(—)	>100
Caffeic acid	1837.28 ± 6.32	8.57 ± 0.54	(—)	(—)	32.26 ± 0.23
Syringic acid	1329.10 ± 5.37	10.25 ± 0.35	(—)	(—)	>100
Epicatechin	1257.56 ± 3.59	20.72 ± 1.23	(—)	(—)	>100
BHT	321.36 ± 2.56	103.45 ± 3.89	(—)	(—)	(—)
Indomethacin	(—)	(—)	(—)	(—)	74.36 ± 0.12

*Values represented mean ± S.D. of three parallel measurements (*P* < 0.05).

^a^Data expressed in *μ*g catechin equivalent/mg dry weight (mg CE/g).

^b^Data expressed in *μ*g rutin equivalent/mg dry weight (mg RE/g).

**Table 2:** Effects of EAAC and indomethacin (Indo) on changes in CAT, SOD, and GPx activities were studied in Carr-induced paw edema (5th hour) in mice.

Groups	Catalase (U/mg protein)	SOD (U/mg protein)	GPx (U/mg protein)
Control	5.35 ± 0.09	24.21 ± 0.12	23.43 ± 0.09
Carr	3.39 ± 0.15^###^	13.69 ± 0.07^###^	14.67 ± 0.16^###^
Carr + Indo	4.85 ± 0.36**	22.15 ± 0.10**	20.13 ± 0.19**
Carr + EAAC (62.5 mg/Kg)	3.53 ± 0.06	15.75 ± 0.13	16.85 ± 0.06
Carr + EAAC (125 mg/Kg)	4.21 ± 0.05*	19.07 ± 0.05*	19.06 ± 0.05*
Carr + EAAC (250 mg/Kg)	5.08 ± 0.11**	22.47 ± 0.09**	21.09 ± 0.21**

Each value represents mean ± S.E.M. ^###^ *P* < 0.001 as compared with control group. *P < 0.05 and **P < 0.01 as compared with the Carr (*λ*-carrageenan) group (one-way ANOVA followed by Scheffe's multiple range test).
